# Growth of H5N1 Influenza A Viruses in the Upper Respiratory Tracts of Mice

**DOI:** 10.1371/journal.ppat.0030133

**Published:** 2007-10-05

**Authors:** Masato Hatta, Yasuko Hatta, Jin Hyun Kim, Shinji Watanabe, Kyoko Shinya, Tung Nguyen, Phuong Song Lien, Quynh Mai Le, Yoshihiro Kawaoka

**Affiliations:** 1 Department of Pathobiological Sciences, School of Veterinary Medicine, University of Wisconsin-Madison, Madison, Wisconsin, United States of America; 2 The Avian Zoonosis Research Centre, Tottori University, Tottori, Japan; 3 National Centre for Veterinary Diagnostics, Hanoi, Vietnam; 4 Vietnam Veterinary Association, Hanoi, Vietnam; 5 National Institute of Hygiene and Epidemiology, Hanoi, Vietnam; 6 Institute of Medical Science, University of Tokyo, Tokyo, Japan; 7 Core Research for Evolutional Science and Technology, Japan Science and Technology Agency, Kawaguchi, Saitama, Japan; The Pennsylvania State University, United States of America

## Abstract

Highly pathogenic avian H5N1 influenza A viruses have spread throughout Asia, Europe, and Africa, raising serious worldwide concern about their pandemic potential. Although more than 250 people have been infected with these viruses, with a consequent high rate of mortality, the molecular mechanisms responsible for the efficient transmission of H5N1 viruses among humans remain elusive. We used a mouse model to examine the role of the amino acid at position 627 of the PB2 viral protein in efficient replication of H5N1 viruses in the mammalian respiratory tract. Viruses possessing Lys at position 627 of PB2 replicated efficiently in lungs and nasal turbinates, as well as in cells, even at the lower temperature of 33 °C. Those viruses possessing Glu at this position replicated less well in nasal turbinates than in lungs, and less well in cells at the lower temperature. These results suggest that Lys at PB2–627 confers to avian H5N1 viruses the advantage of efficient growth in the upper and lower respiratory tracts of mammals. Therefore, efficient viral growth in the upper respiratory tract may provide a platform for the adaptation of avian H5N1 influenza viruses to humans and for efficient person-to-person virus transmission, in the context of changes in other viral properties including specificity for human (sialic acid α-2,6-galactose containing) receptors.

## Author Summary

Highly pathogenic avian H5N1 influenza A viruses have spread around the world since 2003, raising serious worldwide concern about their pandemic potential. Although efficient human-to-human transmission of this virus has not yet occurred, the potential of these viruses to acquire the ability is evident. The receptor specificity of the haemmaglutinin (HA) protein is considered a main factor affecting efficient transmission of H5N1 viruses. Yet, some H5N1 viruses isolated from humans that possess human receptor specificity have still failed to spread efficiently among humans. Therefore, amino acid substitutions in viral proteins other than the receptor-binding HA protein must be necessary for efficient growth and person-to-person transmission of avian H5N1 influenza virus. In our study, we defined the contribution of the amino acid at position 627 of the PB2 to efficient replication of H5N1 influenza viruses in the upper respiratory tracts of mice as a mammalian model. Because efficient viral growth in the upper respiratory tract of humans can facilitate virus excretion by coughing and sneezing, a mutation of PB2 amino acid 627, which contributes to efficient growth at this site in a mammal, may be prerequisite for efficient human-to-human transmission.

## Introduction

The first outbreak in humans caused by the highly pathogenic H5N1 influenza A virus was reported in Hong Kong in 1997, and resulted in the deaths of six of 18 infected people [1–3]. This event demonstrated for the first time the direct transmission of a highly pathogenic avian influenza virus from birds to humans with a fatal outcome. In December 2003, this virus began to spread widely in poultry in Vietnam, Indonesia, and Thailand and has since spread to countries in the Middle East, Europe, and Africa, resulting in huge economic losses in the poultry industries of the affected regions. More than 250 human infections have been identified, of which more than 150 have been fatal [4], raising serious worldwide concern about a catastrophic influenza pandemic. Fortunately, efficient human-to-human transmission of this virus has not yet occurred, lending impetus to efforts to identify the molecular mechanisms that might promote the transmission and resulting pandemic strain of this highly pathogenic H5N1 influenza virus.

The receptor specificity of the surface glycoprotein haemmaglutinin (HA) is thought to be one of the determinants for efficient person-to-person transmission of influenza A virus. Avian influenza viruses preferentially recognize receptors with saccharide terminating in sialic acid α-2,3-galactose (SAα2,3Gal) on avian cells, and human viruses preferentially bind to receptors with saccharide ending in sialic acid α-2,6-galactose (SAα2,6Gal) on human cells [5–8]. Indeed, the first human isolates from the 1957 and 1968 pandemics preferentially recognize SAα2,6Gal despite the fact that their HAs were derived from avian viruses. In spite of their preference, or at least partial preference, for SAα2,6Gal [9–11], some of the H5N1 viruses isolated from humans still failed to spread efficiently among humans [9]. Hence, amino acid substitutions in viral proteins other than the HA might be required for the efficient growth and person-to-person transmission of avian H5N1 influenza virus in humans.

To understand why H5N1 influenza virus infection leads to severe pneumonia [12] in humans but to only limited human-to-human transmission [12], we focused on two human H5N1 influenza viruses isolated from the same patient and compared their growth in mice and cells. We defined the contribution of the amino acid at position 627 of the PB2 of H5N1 influenza virus to efficient replication of this virus in the respiratory tracts of mice.

## Results/Discussion

### Differences in the Replication of A/Vietnam/1203/2004 and A/Vietnam/1204/2004 Viruses in Mice

Two H5N1 variants isolated from the same patient in Vietnam in 2004 [13]—A/Vietnam/1203/2004 (VN1203; upper respiratory, pharyngeal swab) and A/Vietnam/1204/2004 (VN1204; lower respiratory tract, tracheal aspirate)—differ by six amino acids (two in PB2, three in PA, and one in NS1; Table S1). We found one of these amino acid differences, position 627 of PB2, highly intriguing. VN1203 possesses Lys at this position (PB2-627Lys), while VN1204 possesses Glu (PB2-627Glu). The vast majority of authentic human influenza A viruses (i.e., H1N1 and H3N2 subtypes) characterized to date have Lys at this position (a few have Arg), and avian isolates generally have Glu (with some exceptions, noted below). The amino acid at position 627 has been associated with the host specificity of influenza A viruses [14–16]. Moreover, PB2-627Lys has been linked to the high virulence of H5N1 viruses in mice [17] and to an H7N7 virus isolated from a human case of fatal influenza in the Netherlands in 2003, although neither the isolate from a patient with conjunctivitis or those from chickens examined during that outbreak had Lys at this position [18]. The amino acid at PB2–627 also affects the efficiency of RNA polymerase activity at a low temperature in certain types of cells [19]. However, no direct experimental evidence demonstrates that the Glu-to-Lys mutation at PB2–627 supports efficient growth of avian influenza viruses in the upper respiratory tracts of mammals or efficient transmission among them. We, therefore, investigated the biologic properties of these two H5N1 isolates in a mouse model, with the intent of assessing the contribution of the PB2–627 amino acid in efficient replication of H5N1 influenza viruses in the human respiratory tract.

The VN1203 isolate possessing PB2-627Lys was slightly more pathogenic in mice than VN1204 possessing PB2-627Glu, as indicated by an MLD_50_ (the dose required to kill 50% of mice) of 0.7 plaque-forming units (PFU), as compared with an MLD_50_ of 2.1 PFU for VN1204 (Table 1). In mice intranasally infected with 100 PFU of virus, VN1203 replicated systemically and VN1204 replicated mainly in respiratory organs. Although both viruses replicated well in the lungs, only VN1203 replicated to a high titer in nasal turbinates in all animals tested (Table 1). To test the role of the amino acid at position 627 of PB2 in replicative capacity, we generated mutants of VN1203 and VN1204 characterized by the following changes at PB2–627: VN1203PB2-627Glu virus possessing Glu at this position in the background of VN1203 and VN1204PB2-627Lys virus possessing Lys in the background of VN1204. When their replication was tested in mice (Table 1), VN1204PB2-627Lys virus was isolated at high titers from a variety of organs, including nasal turbinates (unlike the parent VN1204). VN1203PB2-627Glu was attenuated by two log units (MLD_50_ of 67.6 PFU) as compared with that of wild-type VN1203 (MLD_50_, 0.7 PFU), as expected based on our previous findings [17,20]. VN1203PB2-627Glu generally showed restricted replication in all organs except the lungs, where it replicated as well as wild-type VN1203. In nasal turbinates, the VN1203PB2-627Glu virus was isolated from only one mouse, but from a variety organs in that animal, including the brain at 6 d post-infection. Interestingly, a revertant virus with Lys at position 627 of PB2 was detected in the nasal turbinates and lungs of this animal (unpublished data), suggesting that systemic infection in this mouse might have been caused by the revertant virus. These results indicate that the amino acid at position 627 of PB2 contributed to the efficient growth of the virus in mice, as previously demonstrated [17], and to the efficient replication of this virus in the upper respiratory tract. VN1204PB2-627Lys virus replicated to appreciably higher titers than did VN1203, which also has lysine at PB2–627, suggesting that other amino acid differences between the two viruses may contribute to the increased growth efficiency.

**Table 1:**
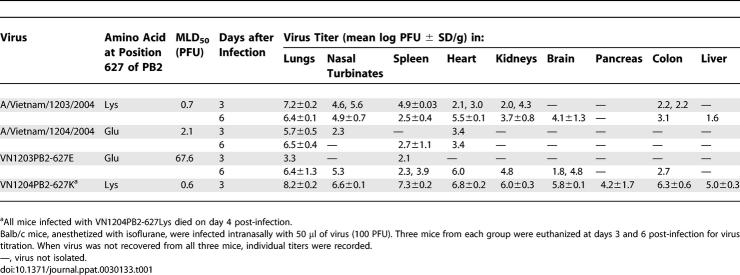
Biological Properties of H5N1 Viruses and Their PB2 Mutants in Mice

### Replication of VN1203 and VN1204 Viruses in Cells at Different Temperatures

**Figure 1:**
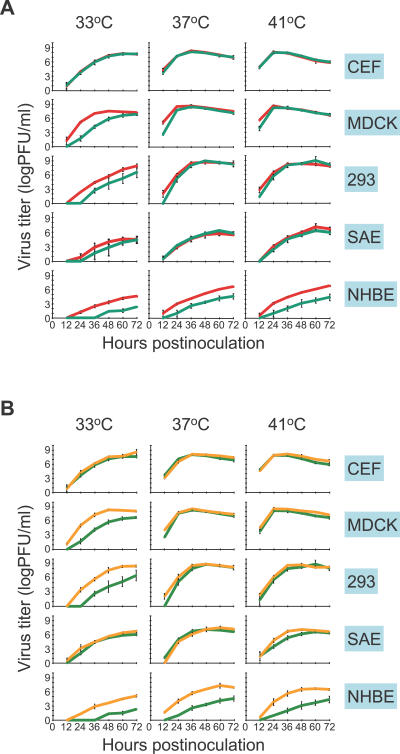
Growth Properties of Viruses at Different Temperatures in Diverse Cell Types (A) VN1203 (red) and VN1204 (green) viruses. (B) VN1204 (green) and VN1204PB2-627Lys (orange) viruses. Cells were infected with virus at a multiplicity of infection of 10^−5^ and incubated at 33, 37, or 41 °C. Aliquots of the supernatants were titrated on MDCK cells by plaque assay. The values are means (± standard deviation) of three independent determinations.

In their 2001 publication, Massin et al. reported that the amino acid at 627 of PB2 affects viral transcription at a low temperature in some cells [19]; hence, the difference in tissue tropism in mice between VN1203 and VN1204 may arise partly from a difference in optimal growth temperatures. To test this hypothesis, we infected cells of different origins at different temperatures and examined the growth properties of VN1203 and VN1204. We found that the viruses replicated equally well in chicken embryo fibroblasts at all temperatures tested (Figure 1A). However, in two mammalian cell lines (Mardin-Darby canine kidney [MDCK] and human embryonic kidney 293 cells), the VN1203 virus replicated more efficiently than did VN1204 at 33 °C, with no substantial differences in their growth found at 37 °C and 41 °C. These results indicate that PB2-627Lys confers a growth advantage to the virus at 33 °C in these cell lines. Viral growth was also compared in two types of human primary cells: small airway epithelia (SAE; representing lung cells) and bronchial/tracheal epithelia (NHBE). At temperatures commonly found in the lower respiratory tract (>37 °C), VN1203 and VN1204 did not show any substantial differences in growth in SAE cells. At 33 °C, the virus with Lys at position 627 replicated slightly better than the Glu variant in these cells, although the standard deviation was too broad to support a significant difference. By contrast, the Lys variant replicated better than the Glu variant in NHBE cells at all temperatures tested.

To prove that the cell type– and growth temperature–dependent difference in replicative ability between VN1203 and VN1204 originated from an amino acid shift at PB2–627, we examined the growth properties of the VN1204 mutant possessing PB2-627Lys (VN1204PB2-627Lys) in these cells, using wild-type VN1204 as the control. This comparison yielded essentially the same results as found for VN1204 versus VN1203 (Figure 1B), demonstrating that a single substitution at PB2–627 does indeed account for the phenotypic difference between VN1203 and VN1204. Hence, the acquisition of this mutation by an avian H5N1 influenza virus would likely promote better replication in a wider range of cell types (as observed by viral replication in NHBE; Figure 1A) and at a lower temperature than found in the upper respiratory tract, possibly allowing the virus to be readily spread by sneezing and coughing.

### Effect of PB2–627 Amino Acid on Growth Properties of Other Influenza A Viruses in Mice

To determine whether the Lys at amino acid position 627 of PB2 enhances growth of other influenza A viruses in the upper respiratory tract, we intranasally inoculated mice with 10^6^ PFU of viruses isolated from a human (A/Memphis/8/88 (H3N2) (Mem/88)) possessing Lys at this position or from birds (A/mallard duck/New York/6750/78 (H2N2) (Mal/NY), A/chicken/Vietnam/NCVD5/2003 (H5N1) (VD5), and A/muscovy duck/Vietnam/NCVD18/2003 (H5N1) (VD18)) possessing Glu. We collected samples from their nasal turbinates and lungs on day 3 post-inoculation for virus titration. The results indicate that all virus strains used in this study replicated in lung, with virus titers ranging from 10^3.1^ to 10^7.5^ PFU/g (Table 2), while their growth properties in nasal turbinates differed widely. Human influenza A virus Mem/88 possessing PB2-627Lys was recovered at 10^6.1^ PFU/g from this source, but two of the avian influenza viruses (Mal/NY and VD5, both possessing PB2-627Glu) were not detected. Another avian influenza virus VD18 possessing PB2-627Glu was, however, detected in nasal turbinates at relatively high titers. To determine if the efficient replication of VD18 in nasal turbinates originated from the emergence of a mutant virus possessing the Glu-to-Lys mutation at PB2–627, we molecularly cloned the PB2 genes of viruses from animals infected with VD18 (Table 3). Interestingly, a substantial population of a mutant VD18 virus, which possesses an amino acid mutation from Glu to Lys at position 627 of the PB2, was found in nasal turbinates and lung tissues. In addition, the virus titers in the nasal turbinates were correlated with the prevalence of a mutant virus with PB2-627Lys; virus titers in this organ were high in mice (mouse 1 and 3) when the prevalence of PB2-627Lys variant was also high, while the virus titer was low when the prevalence of PB2-637Lys variant was also low (mouse 2; Table 3). This finding further indicates that PB2-627Lys is associated with the efficient growth in upper respiratory tracts.

**Table 2:**
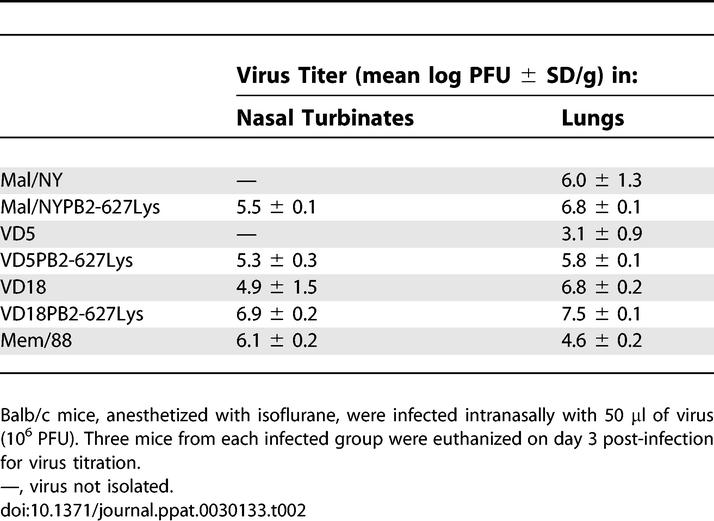
Effect of PB2 Mutation on Virus Titers in Nasal Turbinates and Lungs

**Table 3:**
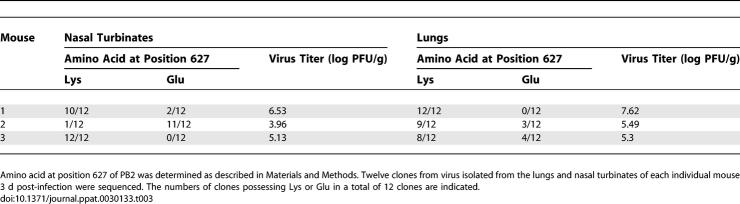
Amino Acid at Position 627 of the PB2 of VD18 (A/chicken/Vietnam/NCVD18/2003) Virus Isolated from Mouse Organs

Next, we generated mutants of the avian viruses, converting Glu to Lys at PB2–627 by reverse genetics (Mal/NYPB2-627Lys, VD5PB2-627Lys, and VD18PB2-627Lys), and examined their growth properties in mice. Although Glu-to-Lys mutation at PB2–627 enhanced replication of Mal/NY and VD5 in lungs, the effect was more dramatic in nasal turbinates (see Mal/NYPB2-627Lys and VD5PB2-627Lys in Table 2). The effect of the Glu-to-Lys mutation at PB2–627 in viral replication in nasal turbinates was not as dramatic with the VD18 virus as that observed with Mal/NY and VD5 viruses. Even so, the mutation enhanced viral replication in nasal turbinates by two log units, while the increase of virus titers in lungs was fewer than one log unit (Table 2). This less dramatic enhancement of viral replication in nasal turbinates by PB2-627Lys mutation in VD18 likely originates from increased virus replication caused by PB2-627Lys variant that naturally emerged in mice infected with VD18 (Table 3). We conclude that Lys at position 627 of PB2 confers to some influenza A viruses the ability to replicate efficiently in the upper respiratory tracts of mice and suggest that it might also enhance the transmissibility of these viruses among humans.

We show here that a mutation in the PB2 protein, from Glu to Lys at position 627, can expand the cell tropism of H5N1 avian influenza A viruses (see results of viral replication in NHBE; Figure 1A) and enable them to grow at a lower temperature. This mutation, together with HA mutations that confer viral SAα2,6Gal recognition [9], could promote the replication of avian influenza viruses in the upper respiratory tracts of humans, although other amino acid substitutions are likely needed to confer full pandemic potential. Indeed, multiple amino acid changes have been identified in the so-called Spanish influenza virus, which is thought to be derived from an avian antecedent [21–23], as compared with the consensus sequences of avian viruses [24–26]. We find it worrisome, in this context, that some H5N1 isolates from humans recognize SAα2,6Gal in addition to SAα2,3Gal [9,10]. Moreover, unlike other avian influenza viruses, H5N1 viruses isolated from wild waterfowl and their descendants during the May 2005 Qinghai Lake outbreak in China possess PB2-627Lys [27,28], suggesting that the acquisition of such mutations may provide a platform for evolution to a pandemic status. Furthermore, we found that PB2-627Glu mutants of VD18 wild-type and VN1203PB2-627Glu viruses, but not those of VN1204, VD5, and Mal/NY viruses, mutated to Lys during replication in mice. This variation among viral strains suggests that other amino acid differences in this or other viral proteins may contribute to the extent of Glu-to-Lys mutation at this position of the PB2. Thus, to acquire the capacity for efficient human-to-human transmission, H5N1 avian influenza A viruses likely must undergo a series of genetic changes resulting in the ability to replicate at lower temperatures and in a wider range of cell types (a trait controlled by the PB2 protein), to recognize human sialyloligosaccharide receptors (a trait controlled by HA molecules), and other unknown phenotypic changes controlled by other viral proteins.

## Materials and Methods

### Viruses.

The highly pathogenic H5N1 viruses used in this study (A/Vietnam/1203/2004 (VN1203), A/Vietnam/1204/2004 (VN1204), A/chicken/Vietnam/NCVD5/2003 (VD5), and A/muscovy duck/Vietnam/NCVD18/2003 (VD18)) were kindly provided by the Centers for Disease Control and Prevention. Human isolates were grown in MDCK cells maintained in minimal essential medium with 5% newborn calf serum. Duck isolates were amplified in 10-d-old embryonated chicken eggs. All experiments with live viruses and with transfectants generated by reverse genetics were performed in a biosafety level 3 containment laboratory approved for such use by the Centers for Disease Control and Prevention and the US Department of Agriculture.

### Cells.

Human embryonic kidney 293 and 293T cells (a derivative of the 293 line into which the gene for simian virus 40 T antigen was inserted) were maintained in Dulbecco's modified Eagle's medium with 10% fetal calf serum and antibiotics at 37 °C with 5% CO_2_. Chicken embryo fibroblasts were prepared from 10-d-old embryonated eggs and maintained in Dulbecco's modified Eagle's medium supplemented with 10% fetal calf serum. Human primary SAE and human primary NHBE cells were purchased from Cambrex Corporation (http://www.cambrex.com/) and maintained at 37 °C with 5% CO_2_, according to the manufacturer's manual.

### Virus growth kinetics in cell culture.

Cells were infected with virus at a multiplicity of infection of 10^−5^ and incubated at 33, 37, or 41 °C. Aliquots of the supernatants were collected at 12, 24, 36, 48, 60, and 72 h post-infection and titrated on MDCK cells by plaque assay.

### Mouse experiments.

Four- to six-wk-old Balb/c mice (The Jackson Laboratory, http://www.jax.org/) were used for the experiments. The dose lethal to 50% of mice (MLD_50_) was determined as previously described [29]. For virus titration in organs, mice were infected intranasally with 100 PFU of virus and euthanized on days 3 and 6 post-infection for virus titration. For the experiments using A/Memphis/8/88 (H3N2) (Mem/88), A/mallard duck/New York/6750/78 (H2N2) (Mal/NY), VD5, and VD18 viruses, all of which were generated by reverse genetics, the mice were intranasally inoculated with 10^6^ PFU of viruses and euthanized on day 3 post-infection. The virus titers in nasal turbinates and lungs were assayed by plaque assays.

### Plasmid construction and reverse genetics.

The cDNAs of the VN1203, VN1204, VD5, and VD18 viruses were synthesized by reverse transcription of viral RNA with an oligonucleotide (Uni 12) complementary to the conserved 3' end of viral RNA, as previously described [17]. The cDNA was amplified by PCR with gene-specific oligonucleotide primers and then sequenced. The generation of plasmid constructs for viral RNA production (pPolI), containing the genes of VN1203, VN1204, VD5, and VD18 viruses flanked by the human RNA polymerase I promoter and the mouse RNA polymerase I terminator, is described in a previous publication [30]. The mutant PB2 pPolI constructs possessing a mutation with amino acid at position 627 of PB2 (VN1203PB2-627Glu, VN1204PB2-627Lys, Mal/NYPB2-627Lys, VD5PB2-627Lys, and VD18PB2-627Lys) were produced by PCR amplification with the primers possessing mutations (primer sequences will be provided upon request). All constructs were sequenced to ensure the absence of unwanted mutations. Automated sequencing was performed at the University of Wisconsin-Madison Biotechnology Center. The reverse genetics systems for Mem/88 and Mal/NY viruses are described in previous reports [31]. The production of all transfectant viruses by reverse genetics was performed as described by Neumann et al [30].

### Sequence analysis of the viruses in mouse organs.

RNA was extracted from the supernatants of organ homogenates from infected mice using the RNeasy Mini Kit (Qiagen, http://www.qiagen.com/). The cDNAs were synthesized by reverse transcription of viral RNA with an oligonucleotide (Uni 12) complementary to the conserved 3' end of viral RNA, as described above. The cDNA was amplified by PCR with gene-specific oligonucleotide primers for the PB2 fragment of nucleotide region 1260 to 2341 and cloned into pSTBlue-1 AccepTor or pT7Blue blunt vector (EMD Biosciences, http://www.emdbiosciences.com/). Twelve clones of each virus were sequenced.

## Supporting Information

Table S1 Amino Acid Differences between A/Vietnam/1203/2004 and A/Vietnam/1204/2004 Viruses (31 KB DOC)

### Accession Numbers

The Influenza Sequence Database (http://www.flu.lanl.gov/) accession numbers for the genes described in this paper are ISDN242716 (A/chicken/Vietnam/NCVD5/2003, PB2 gene), ISDN242717 (A/chicken/Vietnam/NCVD5/2003, PB1 gene), ISDN242718 (A/chicken/Vietnam/NCVD5/2003, PA gene), ISDN242719 (A/chicken/Vietnam/NCVD5/2003, HA gene), ISDN242720 (A/chicken/Vietnam/NCVD5/2003, NP gene), ISDN242721 (A/chicken/Vietnam/NCVD5/2003, NA gene), ISDN242722 (A/chicken/Vietnam/NCVD5/2003, M gene), ISDN242723 (A/chicken/Vietnam/NCVD5/2003, NS gene), ISDN242724 (A/muscovyduck/Vietnam/NCVD18/2003, PB2 gene), ISDN242725 (A/muscovyduck/Vietnam/NCVD18/2003, PB1 gene), ISDN242726 (A/muscovyduck/Vietnam/NCVD18/2003, PA gene), ISDN242727 (A/muscovyduck/Vietnam/NCVD18/2003, HA gene), ISDN242728 (A/muscovyduck/Vietnam/NCVD18/2003, NP gene), ISDN242729 (A/muscovyduck/Vietnam/NCVD18/2003, NA gene), ISDN242730 (A/muscovyduck/Vietnam/NCVD18/2003, M gene), ISDN242731 (A/muscovyduck/Vietnam/NCVD18/2003, NS gene).

## Abbreviations

HA: haemmaglutinin
MDCK: Mardin-Darby canine kidney
MLD_50_: the dose required to kill 50% of mice
NHBE: bronchial/tracheal epithelia
PFU: plaque-forming unit
SAα2,3Gal: sialic acid α-2,3-galactose
SAα2,6Gal: sialic acid α-2,6-galactose
SAE: small airway epithelia
