# Supplementary material for: Growth of H5N1 Influenza A Viruses in the Upper Respiratory Tracts of Mice
Source: PLoS Pathog. 2007 Oct 5;3(10):e133. doi: 10.1371/journal.ppat.0030133 (PMC2000968; doi:10.1371/journal.ppat.0030133)
Supplement: Supplementary file 1 [file ppat.0030133.st001.doc]

| **Supporting information (SI) Table S1.** **Amino Acid Differences Between A/Vietnam/1203/2004 and A/Vietnam/1204/2004 Viruses** | | | |
| --- | --- | --- | --- |
|  | Amino acid position | Amino acid | |
|  |  | A/Vietnam/1203/2004 | A/Vietnam/1204/2004 |
| PB2 | 456 | Asn | Asp |
|  | 627 | Lys | Glu |
| PA | 85 | Thr | Met |
|  | 142 | Glu | Lys |
|  | 615 | Lys | Glu |
| NS1 | 82 | Ser | Pro |
